# Examination of spotted sand bass (*Paralabrax maculatofasciatus*) pollutant bioaccumulation in San Diego Bay, San Diego, California

**DOI:** 10.7717/peerj.213

**Published:** 2013-11-19

**Authors:** Chad L. Loflen

**Affiliations:** California Water Quality Control Board – San Diego Region, San Diego, CA, USA

**Keywords:** Bioaccumulation, San Diego Bay, Spotted sand bass, PCBs, Mercury

## Abstract

The spotted sand bass (*Paralabrax maculatofasciatus*) is an important recreational sport and subsistence food fish within San Diego Bay, a large industrialized harbor in San Diego, California. Despite this importance, few studies examining the species life history relative to pollutant tissue concentrations and the consumptive fishery exist. This study utilized data from three independent spotted sand bass studies from 1989 to 2002 to investigate PCB, DDT, and mercury tissue concentrations relative to spotted sand bass age and growth in San Diego Bay, with subsequent comparisons to published pollutant advisory levels and fishery regulations for recreational and subsistence consumption of the species. Subsequent analysis focused on examining temporal and spatial differences for different regions of San Diego Bay.

Study results for growth confirmed previous work, finding the species to exhibit highly asymptotic growth, making tissue pollutant concentrations at initial take size difficult if not impossible to predict. This was corroborated by independent tissue concentration results for mercury, which found no relationship between fish size and pollutant bioaccumulation observed. However, a positive though highly variable relationship was observed between fish size and PCB tissue concentration.

Despite these findings, a significant proportion of fish exhibited pollutant levels above recommended state recreational angler consumption advisory levels for PCBs and mercury, especially for fish above the minimum take size, making the necessity of at-size predictions less critical. Lastly, no difference in tissue concentration was found temporally or spatially within San Diego Bay.

## Introduction

The spotted sand bass (Serranidae) is one of three temperate *Paralabrax* basses in southern California that are target species for recreational and subsistence anglers (California Department of Fish and Game, 2004; Erisman et al., 2011). Protected from commercial fishing in the United States since 1953, the spotted sand bass has a historic range from Mazatlan, Baja California, to Monterey in Central California. The spotted sand bass is generally restricted to shallow protected waters, inhabiting bays, lagoons, and estuaries with a permanent tidal connection and sufficient nursery habitat, including San Diego Bay (Allen et al., 1995).

San Diego Bay is a highly urbanized semi-enclosed bay located along the southwestern coast of the United States. Despite draining a watershed of over 2300 km^2^, San Diego Bay is predominantly saline due to limited freshwater inputs (Largier, Hollibaugh & Smith, 1997). The bay only receives periodic freshwater inflows immediately following storm events due to average annual precipitation of 25–33 cm combined with significant upstream impoundments (SDRWQCB, 1994). The southern portion of the bay experiences hypersaline conditions and higher temperatures due to shallow waters and residence times of over 30 days, whereas the northern portion of the bay is driven by semi-diurnal tides and extensive dredging for maritime purposes (Largier, 1995). The area surrounding the bay has been built out by development, and includes numerous shipyards, military installations, industrial production areas (e.g., aircraft), and high density residential and commercial buildings. Prior to the implementation of the Clean Water Act, waste discharges were released directly to the bay, without treatment, from factories, power stations, industrial plants, and shipbuilding operations (Terzich, 1965). These untreated discharges resulted in anoxic conditions, particularly in areas of the inner bay with high residence times (California. San Diego Regional Water Pollution Control Board, 1952). While waste discharges to San Diego Bay have largely ceased or receive treatment pursuant to environmental regulation, legacy pollutants remain in San Diego Bay sediments, including polychlorinated biphenyls (PCBs), polynuclear aromatic hydrocarbons (PAHs), pesticides, and metals (Fairey et al., 1998; SDRWQCB, 2012). The entire bay is currently listed on the Clean Water Act section 303(d) list as impaired for PCBs (SDRWQCB, 2012). These pollutants have been found to be bioavailable and bioaccumulate within various organismal trophic levels in San Diego Bay (Deheyn & Latz, 2006; Komoroske et al., 2011; Komoroske et al., 2012).

Although protected from commercial fishing, the spotted sand bass is subject to fishing pressure from recreational and subsistence fisheries within San Diego Bay. While the subsistence fishery for San Diego Bay does not exclusively target a species (SDCDHS, 1990), the recreational fishery is largely species specific and focused on capture and release for sport (Leet et al., 2001). Despite its importance as a Southern California fishery, spotted sand bass ecology is not as well documented as for other Serranidae, including *Paralabrax clathratus* and *Paralabrax nebulifer*. Allen et al. (1995) examined the life history of spotted sand bass in the Southern California bight, including age, growth, maturation, reproduction, sex composition, feeding, and recruitment. The study included sampling of fish in San Diego Bay, and found the species to exhibit rapid growth during the first two years following settlement. However, the study is limited in some respects for the bioaccumulative management of the fishery, as growth models had limited samples for older fish and did not differentiate between sexes. For pollutants that bioaccumulate over time, such as mercury, growth modeling can be utilized by fishery managers to predict at-age tissue concentrations, and advise the public regarding consumption (McMurtry et al., 1989; Barber, Suarez & Lassiter, 1991; Storelli, Giacominelli-Stuffler & Marcotrigiano, 2006). Some fish species also exhibit spatial and temporal differences in bioacummulation due to physical conditions (e.g., salinity and temperature), migratory life histories, and/or changes in dietary composition (Boudou et al., 1979; Eagles-Smith et al., 2008; Farmer, Wright & DeVries, 2010; Mousavi et al., 2011).

This study used data from three independent reports on San Diego Bay for spotted sand bass (Table 1) to examine the species life history relative to pollutant tissue concentrations and the consumptive fishery. Differences in bioaccumulation were also explored spatially and temporally for San Diego Bay. Two of the three reports (“1989” and “2002”) include data on fish tissue concentrations, while the third report (“2002 non-tissue”) contains data from the collection of a large sample size of individuals of the species (*n* = 253) with associative information on their size, sex, and age.

**Table 1:** San Diego Bay reports utilized for *Paralabrax maculatofasciatus* analysis.

Year	Fish oversight	Report	Tissue analysis	Sample size (*n*)	Fish	
					Aged	Sexed
1989	Port of San Diego	San Diego Bay Health Risk Study (SDCDHS, 1990)	Yes	15	No	No
2002	State of California	Coastal Fish Contamination Program (Gassel, Brodberg & Roberts, 2002)	Yes	25	No	No
2002	State of California	Necropsy and Histopathology of Spotted Sea Bass Sampled from San Diego Harbor in September 2002 (Marty, 2003)	No	253	Yes	Yes

Analysis of data from the reports was conducted in three steps. First, tissue pollutant concentration data for fish from the 1989 and 2002 reports were used to explore relationships between fish growth and bioaccumulation, with subsequent comparisons to known recreational and subsistence fishing efforts and regulations from California’s Office of Environmental Health Hazard Assessment (OEHHA) and Department of Fish and Wildlife. Following this analysis, data from the 2002 non-tissue report was used to determine if at-age size could be predicted, and if that prediction differed by sex. Lastly, tissue concentrations within San Diego Bay were compared spatially and temporally in consideration of documented pollutant-growth relationships.

## Materials and Methods

### Fish collection

**Figure 1:**
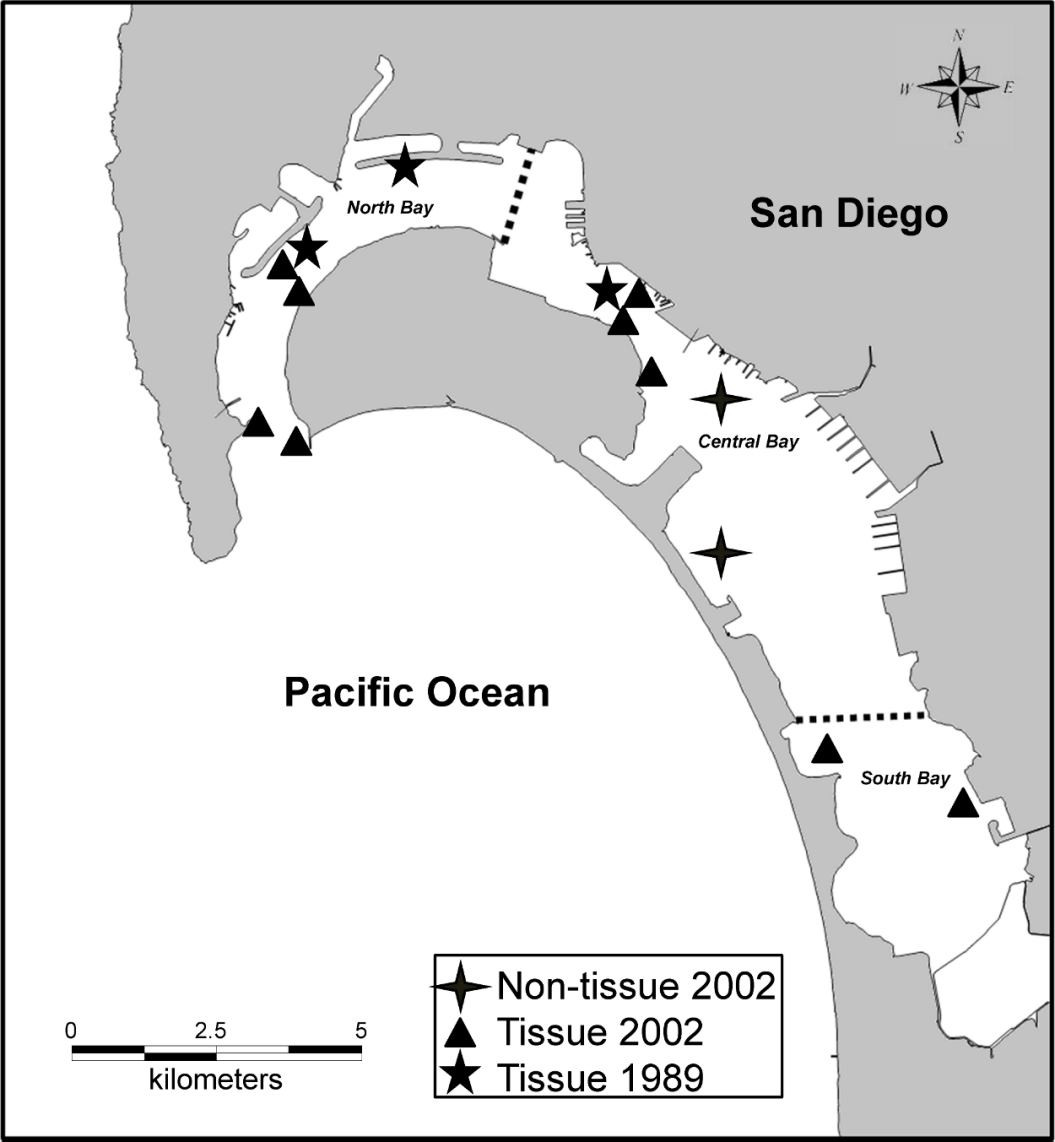
San Diego Bay sampling sites in San Diego, CA, USA (32.66987 N, 117.139521 W).

Spotted sand bass from the 2002 non-tissue report (Marty, 2003) were collected via hook and line from central San Diego Bay (Fig. 1), with a total of 253 fish caught from September 25–29, 2002. The spotted sand bass from the 1989 and 2002 tissue studies were collected in 1989 and from 1999–2002, respectively, via trawls and hook and line within various locations in San Diego Bay (Fig. 1). Fish from the 1989 study were collected by the County of San Diego while the remaining fish were collected beginning 10 years later through the State of California’s Coastal Fish Contamination Program (SDCDHS, 1990; Gassel, Brodberg & Roberts, 2002).

### Necropsy and lab analysis: tissue reports

Fish for tissue analysis were killed by wet ice immersion. Following capture each fish was weighed, measured, and examined for lesions and abnormalities (e.g., fish hooks). Fork lengths (FL) measurements of the Serranidae in southern California are considered equal to total length (TL) by the California Department of Fish and Wildlife’s State Finfish Management Project, and thus the two terms are used interchangeably. Spotted sand bass for tissue analysis were stored on ice and delivered within 24 h for lab processing. Fish not processed within 24 h were frozen until analysis.

Processing and analysis of 1989 report tissues was conducted by Pacific Analytical, Inc., with methods, quality assurance (QA) and quality control (QC) procedures described in detail in SDCDHS (1990), Appendix H. Processing and analysis of 2002 report tissues was conducted by the State of California’s Water Pollution Control Lab and Moss Landing Marine Laboratory, with QA/QC procedures followed as prescribed in a Quality Assurance Program Plan (Ichikiwa, 2000) in accordance with USEPA requirements and recommendations (USEPA (U.S. Environmental Protection Agency), 2000). Fish from each site were frozen at −20 Celsius until dissection. Fish were dissected into fillets and composited into groups, with each composite containing four to six fish. Fifteen composites were prepared in 1989, and twenty-five prepared from 1999 to 2002. The 1989 report tissue composites within each site were created by size class using weight, with the first sample containing the four smallest fish and last containing the four largest. Composites from the 2002 report targeted fish above the historic minimum take size (305 mm length), and were created using USEPA’s 75 percent rule (USEPA (U.S. Environmental Protection Agency), 2000), where the smallest fish was no less than 75 percent of the length of the largest fish in the composite. Fish for tissue analysis were not aged or sexed. 1989 report tissue composites were analyzed for percent moisture and lipid content, DDTs (4,4′ DDE and DDT), PCBs (Aroclor 1254 and 1260), and mercury. 2002 report tissues were analyzed for a suite of pollutants, including all DDTs, total PCBs, and mercury

### Necropsy and lab analysis: non-tissue report

The necropsy procedure for age and sex determination in the 2002 non-tissue study was performed on-vessel in San Diego Bay, with fish held in pumped San Diego Bay seawater for a period no longer than eight hours, though holding times were typically much less. Fish were killed as described in Stehr, Myers & Willis (1994), with minor modifications. Spotted sand bass were killed via an overdose of anesthesia followed by exsanguination during necropsy (Marty, 2003). Tissues for histopathology were then removed as described by Marty (2003) followed by removal of the right otolith. Processing and analysis of 2002 tissues and otoliths for sex and age, respectively, was conducted at the Central Histological Facility in Sacramento, CA, with methods described in detail in Marty (2003) and Marty et al. (1998), Marty et al. (1999).

### Data entry and transformation

All data were entered into Microsoft Excel (Microsoft 2012) and exported to R for data summary and statistical analysis (R Core Team, 2013). Data were log-transformed when necessary to meet test assumptions. Assumptions of normality were evaluated visually while those of homogeneity of variances were evaluated using the F-test. Where assumptions were not met, data was evaluated using alternative statistical methods. For statistical comparisons, fish classified by Marty (2003) as female-intermediate or male-intermediate were analyzed as female and male, respectively, unless otherwise specified (Sadovy & Domeier, 2005).

### Bioaccumulation

As fish collected for tissue analysis were not aged or sexed, for bioaccumulation linear regression was used to investigate relationships between mean composite size, as weight and fork length, to observed tissue pollutant concentrations. Linear regression was then performed for organic pollutants in each composite to evaluate if there was any relationship between organic pollutant bioaccumulation rates. Subsequent analysis was then conducted to determine if differences in bioaccumulation between sites and years for each pollutant occurred using MANOVA. Pollutant concentrations were normalized to body weight or length where necessary based on linear regression results. For analysis sites were pooled to represent different ecoregions within San Diego Bay (see Komoroske et al., 2012), while years were pooled to represent two classes of 1989 and 1999–2002, as the bulk of samples from the 2002 report were collected in 2001. Analysis of PCBs was limited to Aroclors 1254 and 1260 to allow for comparison of 1989 and 2002 tissues. For MANOVA size classes for analysis were restricted to those that overlapped with previous age ranges analyzed. Lastly, mean composite pollutant concentrations were compared to OEHHA published fish tissue goals and tiered advisory levels for recreational anglers (Klasing & Brodberg, 2008) using Bonferroni adjusted *t*-tests.

### Growth modeling

The von Bertalanffy growth curve model was used to develop individual growth curve models for spotted sand bass by sex. Fork lengths by age and body weights were modeled using a common von Bertalanffy growth model (Beverton, 1954; Beverton & Holt, 1957) represented by:

\begin{eqnarray*} \displaystyle E[L\vert t]={L}_{\infty }(1-{e}^{-K(t-{t}_{0})})&&\displaystyle \end{eqnarray*}

where:

*E*[*L*|*t*] = the expected or average length at time (or age) *t*,
*L*_∞_ = the asymptotic average length,
*K* = the so-called Brody growth rate coefficient (units are yr^−1^), and
*t*_0_ = a modeling artifact that is said to represent the time or age when the average length was zero.

Models were developed for each group using the R package as prescribed by Ogle (2011). Subsequent model comparison by groups was conducted by means of ANOVA after testing assumptions of model generalization for variable parameters.

Where von Bertalanffy models could not be fit, relationships were analyzed using linear regression to test for correlation between age and fork length or weight. Fork length data was log-transformed where necessary and to provide a comparison to the findings of Allen et al. (1995).

### Size and weight structure

To examine the size and weight structure of spotted sand bass in San Diego, the 253 individuals from the 2002 non-tissue report were analyzed for differences in mean length and weight by sex utilizing Welch’s *t*-test with Bonferroni corrections for multiple comparisons. Length frequencies for each sex were subsequently compared to the historic legal size limit for the recreational fishery utilizing *t*-tests (305 mm, California Department of Fish and Game, 2012), which best represents the size limit for fish taken during the report time frame.

## Results

### Bioaccumulation

**Figure 2:**
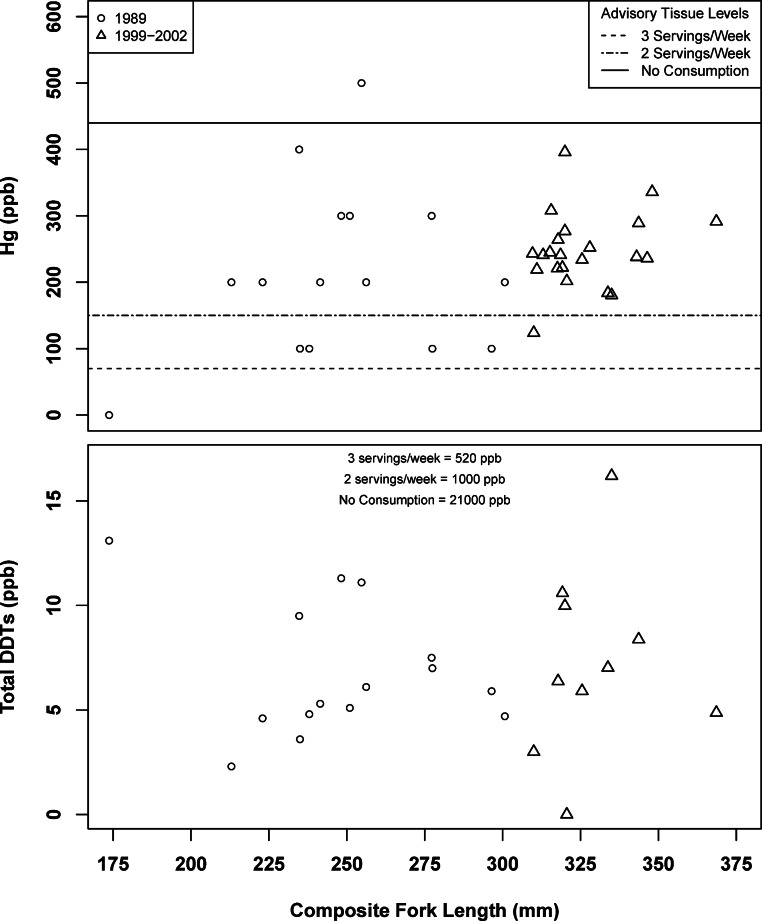
Spotted sand bass (*Paralabrax maculatofasciatus*) tissue concentrations of mercury and DDTs by composite length in San Diego Bay, with comparisons to tissue advisory levels.

**Figure 3:**
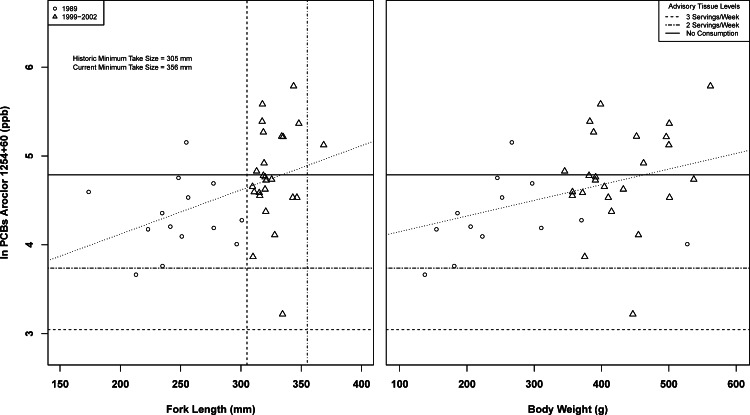
Spotted sand bass (*Paralabrax maculatofasciatus*) PCB tissue concentrations by composite fork length and weight in San Diego Bay, with regression lines.

No relationship was found between composite fish weight or total length and pollutant concentration for mercury or DDTs (Fig. 2). A positive relationship was observed when comparing PCB tissue concentration to body weight (*df* = 1, 37, *F* = 6.65, *r*^2^ = 0.1523, *p* < 0.05, Fig. 3) and fork length (*df* = 1, 37, *F* = 7.14, *r*^2^ = 0.1618, *p* < 0.05, Fig. 3). Organic pollutants were found to be significantly correlated (*df* = 1, 12, *F* = 11.13, *r*^2^ = 0.336, *p* < 0.01). Subsequent MANOVAs were attempted for mercury, body-size adjusted PCBs, and DDTs, with no differences found between ecoregions or between the 1989 and 1999–2002 time periods. There was no evidence of a year: ecoregion interaction. PCB and mercury mean composite tissue concentrations were found to exceed California Advisory Tissue Levels for recreational anglers, and multiple individual composites had tissue concentrations exceeding no consumption thresholds (Table 2, Figs. 2 and 3). Mean DDTs were found to be at least an order of magnitude less than advisory tissue levels, with DDE being the only variant detected.

**Table 2:** *Paralabrax maculatofasciatus*. Comparison of mean composite tissue concentrations to OEHHA Fish Contaminant Goals and Advisory Levels.

Pollutant	Criteria (ppb)^*^	Mean (ppb)	95% CI	*df*	*t*	*p*
Mercury	Goal (220)	233.61	202.43, 264.80	36	5.439	<0.001
	Level 3 (70)^*^					
	Level 2 (150)^*^					
	No consumption (440)					
DDTs^a^	Goal (21)	6.969	5.471, 8.469	24	−19.317	1
	Level 3 (520)					
	Level 2 (1000)					
	No consumption (2100)					
PCBs^a^	Goal (3.6)^*^	100.53	84.26, 119.93	38	10.012	<0.001
	Level 3 (21)^*^					
	Level 2 (42)^*^					
	No consumption (120)					

**Notes.**

* Asterisk denotes significance above criteria, with *t*-test results for the highest level exceeded.

a DDT and PCB criteria, mean, and confidence intervals reported as un-transformed numbers. PCBs analyzed include Aroclors 1254 and 1260.

### Growth models

**Figure 4:**
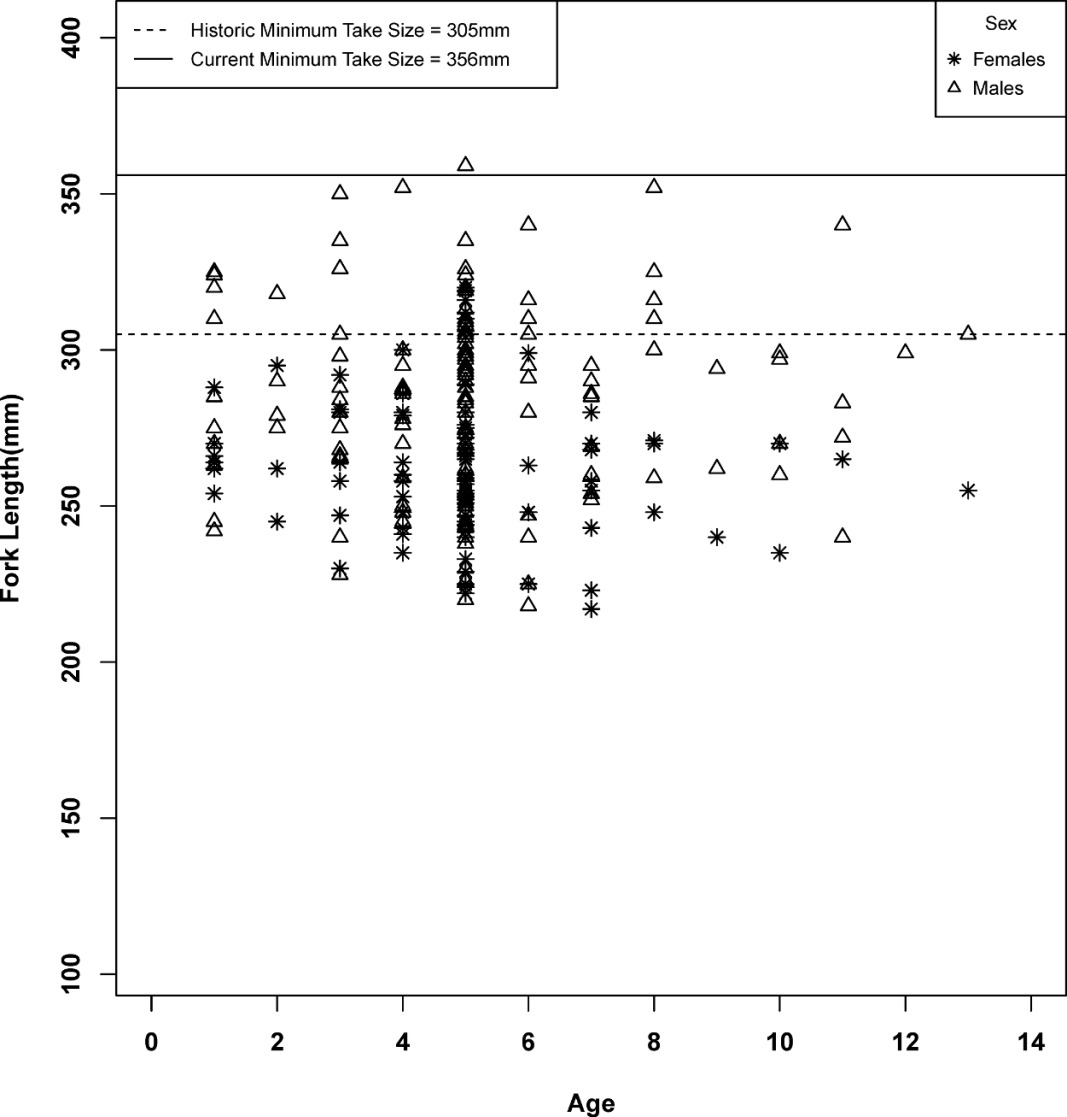
Fork length at age class for male and female spotted sand bass (*Paralabrax maculatofasciatus*).

**Figure 5:**
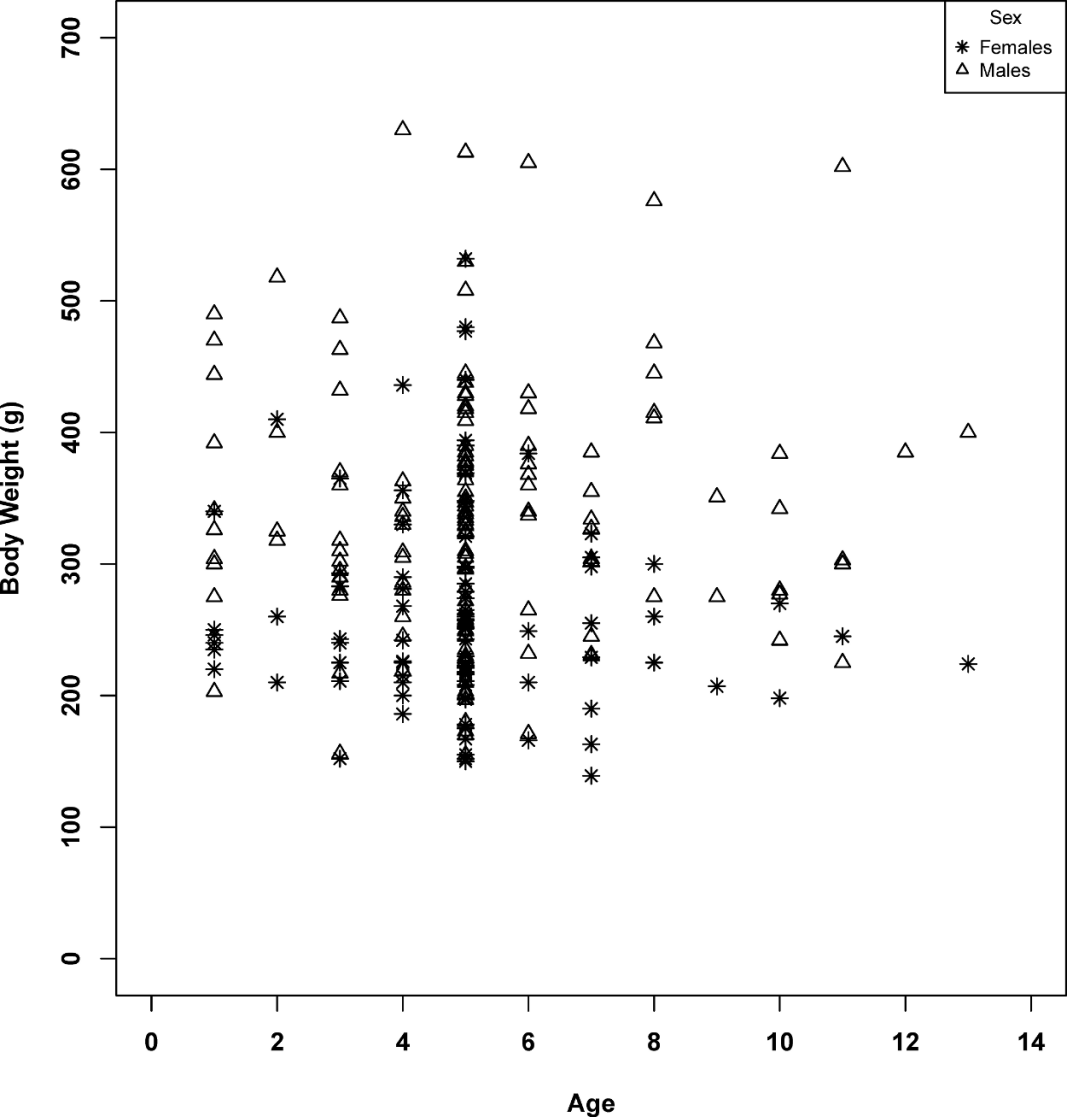
Body weight at age class for male and female spotted sand bass (*Paralabrax maculatofasciatus*).

Growth models were developed for spotted sand bass utilizing age data for observed fork lengths and body weights. Individual von Bertalanffy models for fork lengths and body weights were attempted for each sex. All models developed failed initial assumptions required for modeling, and for those which start data were able to be calculated, individual growth models were unable to be evaluated statistically or run with accuracy due to model failure or exclusion of portions of the dataset to obtain fit. Visual evaluation of age-weight and fork length-weight relationships (Figs. 4 and 5) indicated that primary growth occurred prior to the first year of age, as indicated by the literature, with subsequent age classes showing a consistent range in variability for weight and fork length for each age class, indicating a very fast approach towards asymptotic growth. However, there was high variability within the population and subsequent analysis of groups by linear regression found no evidence of fish age having a relationship with observed fork length or body weight, making at-age take predictions impossible to calculate.

### Size and weight structure

A total of 253 spotted sand bass were sampled for the 2002 non-tissue report. Lengths sampled ranged from 217 to 359 mm fork length (FL), well above the documented lengths of young of year fish (Pondella & Williams, 2009). Fork length differed between the sexes of spotted sand bass, with males having greater lengths, (*df* = 243.331, *t* = 6.157, *p* < 0.0001). Body weight exhibited a similar pattern, with males having a significantly heavier body weight than females (*df* = 235.798, *t* = 6.8352, *p* < 0.0001). Subsequently comparison of mean FL by sex to the 2012 minimum take size for the recreational fishery (30.48 cm TL, California Department of Fish and Game, 2012) found that the mean size of female and male fish were each less than that required for take in the fishery (Female: *df* = 97, *t* = 18.8, *p* < 0.001; Male: *df* = 154, *t* = 9.9, *p* < 0.001, Fig. 4).

## Discussion

The purpose of this study was two-fold. First, three independent reports spanning a decade were used to evaluate how spotted sand bass growth relates to bioaccumulation of pollutants in San Diego Bay, and how those tissue concentrations compare to pertinent fishery regulations and consumption advisory levels. Second, the same bioaccumulation data was used to evaluate how observed tissue concentrations varied spatially and temporally within San Diego Bay.

Most importantly, the bioaccumulation results raise a continued concern regarding human consumption of this species due to observed tissue concentrations of mercury and PCBs. Pollutant concentrations in all adult fish exceeded OEHHA advisory levels, with some fish exceeding no consumption levels for PCBs and mercury. For mercury, which bioaccumulates over extended time periods, no relationship between fish size and pollutant concentration was observed, corroborating independent life-history findings of no relationship between age and size. PCBs were found to have a relationship with body weight and fork length, though inter-composite variability was high. While the lack of a relationship for mercury and the variability in PCBs makes predicting pollutant concentrations at initial take size difficult, the concentrations observed in all fish were of concern for human consumption by recreational anglers, especially for PCBs found in larger fish, which tended to be male. Should higher consumption rates be present for subsistence anglers, then the levels become even more concerning.

Unfortunately it is unclear as to why an increase in PCB concentrations associated with fish size was observed when no relationship between fish age and growth has been documented in past studies (Allen et al., 1995; Andrews et al., 2005) or in this study. It is suggested that future work should focus on individuals in lieu of composites, and collect age, size, and sex data concurrent with tissue analysis. While adults are typically targeted for consumptive use bioaccumulation studies, the smallest size composite sample tested in 1989 was clearly juvenile (170 mm) and had no detected mercury concentration (Figs. 2 and 3). However, this composite had elevated levels of DDTs and PCBs relative to other composites. It is unknown if this is reflective of maternal transmission, feeding habits, nursery habitat type, or other unknown factors.

While both sexes sampled in the 2002 non-tissue report contained individuals above the minimum take size, the take size for the fishery was recently increased by the California Department of Fish and Wildlife to 356 mm (State of California Department of Fish and Wildlife, 2013). Based on this study’s results for PCBs, this new restriction will increase the consumptive risk for the species. In addition, of the 253 fish sampled in the 2002 non-tissue report, only the largest males met or approached the new take size. This may also be of concern as sex may influence the bioaccumulation in fish species. For example, Johnston et al. (2002) found that large predatory male walleye in the Great Lakes had higher pollutant burdens than females. Future bioaccumulation work should examine differences in bioaccumulation between the sexes for legal sized individuals.

There was also no evidence of a spatial difference within the sampled sites. This is an unexpected result as aquaculture work has found spotted sand bass to have low territoriality (Alvarez-Gonzalez et al., 2001), and initial mark-recapture tagging efforts by the California Department of Fish and Wildlife and the Scripps Institution of Oceanography has found individual fish to display short-term site fidelity, with movements of less than 500 m (L Bellquist, pers. comm., 2013). Lastly, there was no evidence for any generational decrease in PCB or mercury concentrations in the thirteen years from 1989 to 2002, a timeframe which represents the maximum lifespan observed in the species. Given the age of the bioaccumulation data, additional compatible sampling is suggested to further determine the persistence of PCBs and mercury within the species and system.

Despite the documented pollutant concentrations, two distinctively positive findings were found from the study efforts. First, a significant proportion of female fish are less than the minimum take size which, absent evidence of fisheries related size suppression, suggests current size limitations are working to protect breeding stock. Second, the level of the legacy pesticide DDT in tissues was significantly below human health criteria values, and all observed DDTs were of the DDE variant. Furthermore, there was a significant correlation between organic pollutant accumulation between PCBs and DDTs, with high levels of PCBs, a legacy pollutant, documented. This suggests that legacy DDTs would be uptaken at similar rates to PCBs, and that the pollutant may no longer be of major concern at this trophic level in the system.

## Additional Information and Declarations

### Field Study Permissions

The following information was supplied relating to ethical approvals (i.e., approving body and any reference numbers): The County of San Diego collected the data and obtained the scientific collection permits required by the California Department of Fish and Wildlife in 1989–90.
